# A spiral model of musical decision-making

**DOI:** 10.3389/fpsyg.2014.00320

**Published:** 2014-04-22

**Authors:** Daniel Bangert, Emery Schubert, Dorottya Fabian

**Affiliations:** School of the Arts and Media, UNSWSydney, NSW, Australia

**Keywords:** music performance, decision-making, intuitive, deliberate, expertise, dual-process theories, default-interventionist

## Abstract

This paper describes a model of how musicians make decisions about performing notated music. The model builds on psychological theories of decision-making and was developed from empirical studies of Western art music performance that aimed to identify intuitive and deliberate processes of decision-making, a distinction consistent with dual-process theories of cognition. The model proposes that the proportion of intuitive (Type 1) and deliberate (Type 2) decision-making processes changes with increasing expertise and conceptualizes this change as movement along a continually narrowing upward spiral where the primary axis signifies principal decision-making type and the vertical axis marks level of expertise. The model is intended to have implications for the development of expertise as described in two main phases. The first is movement from a primarily intuitive approach in the early stages of learning toward greater deliberation as analytical techniques are applied during practice. The second phase occurs as deliberate decisions gradually become automatic (procedural), increasing the role of intuitive processes. As a performer examines more issues or reconsiders decisions, the spiral motion toward the deliberate side and back to the intuitive is repeated indefinitely. With increasing expertise, the spiral tightens to signify greater control over decision type selection. The model draws on existing theories, particularly Evans’ (2011) Intervention Model of dual-process theories, Cognitive Continuum Theory Hammond et al. (1987), Hammond (2007), Baylor’s (2001) U-shaped model for the development of intuition by level of expertise. By theorizing how musical decision-making operates over time and with increasing expertise, this model could be used as a framework for future research in music performance studies and performance science more generally.

## INTRODUCTION

In performance studies, the topic of decision-making is crucial to understanding how practitioners create and interpret. For example, to perform notated music, musicians make many decisions about stylistic and expressive nuances that may or may not be notated and are likely to vary between performers and performances. This paper seeks to understand and model how performers decide to create such nuances by considering one possible distinction between types of decision-making, namely the difference between intuitive and deliberate decision-making process.

These two types of decisions are defined using a theoretical framework from dual-process theories of cognition. These theories contrast System 1 and 2 (e.g., Kahneman, 2011) or Type 1 and 2 thinking (e.g., Evans, 2011; Stanovich, 2011; Evans and Stanovich, 2013); processes we refer to as intuition and deliberation. Evans (2011) has defined the former (Type 1) as “fast, high capacity, independent of working memory and cognitive ability” and the latter (Type 2) as “slow, low capacity, heavily dependent on working memory and related to individual differences in cognitive ability” (p. 87). The decisions discussed in this paper relate to music performance and are defined as changes to performance features that are reported or observed using empirical methods. In addition, our focus is the processes of decision-making, rather than their results such as decision accuracy or content. Therefore, the model introduced in this paper refers to how changes are made to performance features and whether these processes exemplify Type 1 or Type 2 thinking.

Some research has already shed light on how both intuition and deliberation are used by musicians. For example, Hallam (1995a,b) interviewed twenty-two performers about their practice habits and found differences between those who were “intuitive/serialists” versus “analytic/holists.” Seven musicians preferred the intuitive approach, allowing their interpretation to evolve unconsciously and avoiding deliberate analysis and planning. Two musicians were classed as analytic and relied on deliberate, conscious analysis of the piece. Ten musicians used a mixed, “versatile” strategy, adopting the two approaches interchangeably and three musicians remained unclassified. Overall, Hallam (1995a) found that professional performers “have a tendency to prefer versatile or intuitive/serialist styles of learning” (p. 121).

This paper aims to provide a context for such results by considering the interaction between intuition and deliberation and how their use might change. Hallam’s study implies that many musicians shift between strategies, but what causes such movement and could it be modeled? This paper introduces a spiral model of musical decision-making by summarizing relevant existing models and drawing on recent empirical research about the performance of Western art music. We will argue that our model incorporates the long and short-term development of decision-making and expertise along the intuitive-deliberate continuum that is largely absent from current literature.

## EXISTING MODELS

A number of existing models have attempted to address the relationship between intuition and deliberation. We use these as a starting point for understanding how intuitive and deliberate processes of decision-making operate over time. The models share a number of principles, including: (a) decision-making processes change over time and with increasing expertise; (b) both intuitive and deliberate processes can be employed, but intuition occurs before deliberation; and (c) the nature and content of intuitions can change (future intuitions are informed by analytical thinking).

### THE INTERVENTION MODEL

Among dual-process theories, a common view of the interaction between Type 1 and Type 2 processes are models known as default-interventionist. These models propose that Type 1 processes generate a default response and this response will be given unless there is later intervention by Type 2 processes (e.g., Stanovich, 1999; Kahneman and Frederick, 2002; Evans, 2007). The Intervention Model (Evans, 2011) is a “new general model of intervention on default intuitive responses” (p. 95) that attempts to give a more detailed account of factors that lead to the intervention of Type 2 processes. Evans (2011) proposes that “Type 2 processing is engaged with a *variable* degree of effort” (p. 95) and that motivational factors and cognitive resources influence the degree of effort engaged. The motivational factors that influence the intervention of Type 2 processes include a feeling of rightness (FOR) that accompanies a Type 1 response (Thompson, 2009). According to Thompson, the strength of the FOR determines the probability and extent to which Type 2 processes are engaged to analyze or rethink Type 1 decisions. While a weak FOR should engage a Type 2 response and a strong FOR should retain a Type 1 response, individual differences in monitoring, control and thinking disposition are just some factors that may alter these predicted outcomes. Mangan (1993) has previously suggested that strong feelings of rightness are central to esthetic experience:

Esthetic phenomenology appears to have at its core an especially intense experience of rightness. It is this feeling that gives esthetic experience its phenomenological profile: the sense of immediate correctness, of an especially well-integrated or “right” relation of parts, of a primary and metaphysical YES! of cognitive disclosure (p. 97).

According to the Intervention Model (Evans, 2011), if Type 2 processing is engaged, the initial intuitive Answer 1 (A1) will either be deliberately justified or not. If A1 is not justified, an attempt will be made to rethink the problem and lead to Answer 2 (A2). Evans points out that A1 may actually be the same as A2 and such cases reflect confabulations or justifications for the initial intuition. The attempt to rethink the problem may fail if it is constrained by cognitive resources, resulting in a reversion to the initial A1, a random guess, or no answer altogether.

### COGNITIVE CONTINUUM THEORY

A different explanation is the spectrum of cognition known as Cognitive Continuum Theory (CCT) proposed by Hammond et al. (1987). According to CCT, “the mode of cognition used on any type of judgment, decision-making, or problem solving task can vary on a continuum ranging from intuition to analysis” (Hamm, 1988, p. 758). Intuitive processing is defined as low cognitive control, rapid data processing and low conscious awareness, while analytical processing involves high cognitive control, slow data processing and high conscious awareness (Hammond et al., 1987). Hammond (2007) has acknowledged that the concept of a cognitive continuum is an abstraction; “no one has ever seen such a continuum and, of course, no one ever will” (p. 125–126). He has also explained how cognitive activity can oscillate between intuition and analysis:

In short, the tactics most of us use most of the time are neither fully intuitive nor fully analytical: they are a compromise that contains some of each; how much of each depends on the nature of task and on the knowledge the person making the judgment brings to the task. And over time, the person will move his or her cognitive activity across the cognitive continuum (p. 237).

As well as the notion of a cognitive continuum, Hammond (2007) has outlined a task continuum and a surface-depth continuum that help to contextualize and explain intuition and analysis. The surface-depth continuum refers to data that are close to the person making the judgments (surface) and data about objects and events that are remote in time or place from that person (depth). The task continuum differentiates judgment tasks according to whether they are intuition-inducing or analysis-inducing, with common sense-inducing falling between the two poles. Hammond suggests researchers list properties of a task, locate it on the task continuum, and predict the nature of the judgment that the task induces. This procedure was followed in a study of highway engineers in which judgments and tasks were assessed using a Cognitive Continuum Index (CCI) and Task Continuum Index (TCI) respectively (Hammond et al., 1997). The study required judgments of highway esthetics (intuition-inducing), safety (quasirationality-inducing) and capacity (analysis-inducing), and found better performance when the mode of cognition used (CCI) corresponded to the task properties (TCI).

An earlier study of highway engineers by Hamm (1988) also required judgments of highway esthetics, safety, and capacity, but demonstrated the dynamic nature of the cognitive continuum by mapping analytic and intuitive cognitive activity over time using a Moment by Moment Cognitive Continuum Index (MBMCCI). Hamm suggested five possible ways in which cognitive activity may change: a linear progression from intuitive to analytic thinking or vice versa, non-linear alternation between intuition and analysis or vice versa, and rapid repeated alternation between intuition and analysis. The study found no evidence for linear trends, but there was evidence of alternation between intuition (I) and analysis (A), either in a curve moving from intuition to analytic to intuition (I-A-I) or from analytic to intuition to analytic (A-I-A).

### U-SHAPED DEVELOPMENTAL PROGRESSION OF INTUITIVE THINKING

Baylor (2001) has proposed that intuition matures with increasing levels of expertise in a U-shaped manner. According to this model, intuitive processes are frequently employed by novices with less-developed knowledge structures and by experts with highly developed knowledge structures. The use of deliberation and metacognitive strategies in becoming an expert develops a type of intuition Baylor labels “mature intuition.” In contrast, the intuitions of a novice are qualitatively different and can be considered “immature intuition.” This creates a U-shaped progression of intuitive thinking where the availability of immature intuition is high for a novice, decreases during an intermediate stage, and then climbs to high availability of mature intuition for an expert.

Baylor (2001) states that “the “available intuition” within a given subject area refers to the potentiality for intuitive thinking to exist at a given point in development of an individual’s level of expertise” (p. 239). In the first half of the curve, an intuitive understanding in the form of immature intuition moves to a more analytical and less intuitional understanding. In support of this shift, Baylor cites various empirical studies, including work by Bamberger (1982) on musical intelligence showing that children appear to lose a figural grasp of phrases when they first develop a formal understanding of musical notation. With further development, both components become refined and are used interactively by accomplished musicians. Baylor interprets this finding as an immature understanding moving to an intermediate stage and then progressing to maturity. The intermediate stage is similar to Dreyfus and Dreyfus’ (1986) characterization of the “proficient performer” as a person who intuitively organizes and understands their task, but still finds themselves “thinking analytically about what to do” (p. 29). The “proficient performer” is the mid-point in their influential five-stage model of skill acquisition, consisting of novice, competent, proficient, expert, and master (Dreyfus and Dreyfus, 1980).

In the second half of Baylor’s curve, a person may relinquish some metacognitive control over reasoning and access more mature intuition based on new knowledge structures. A mature type of intuition is also implied in the work of Hogarth (2001), who suggests that intuition can be educated by learning from experience and developing skills through observation, speculation, testing, and generalization (p. 214–247).

Having briefly summarized various theories relevant to the distinction between Type 1 and Type 2 decision-making processes, we will now consider recent empirical studies of music performance to determine how existing models apply to musicians.

## INTUITIVE AND DELIBERATE MUSICAL DECISION-MAKING

Research related to processes of musical decision-making includes the work of Hallam (1995a,b) on different styles of practice discussed earlier and studies by Chaffin in collaboration with performers such as Imreh, Ginsborg, and Lisboa (e.g., Chaffin et al., 2002; Chaffin and Lisboa, 2009; Ginsborg and Chaffin, 2011). Chaffin’s research has shown that while performing, musicians pay deliberate attention to certain specific musical aspects (performance cues) and also have spontaneous performance thoughts. This section will discuss further developments in this area of investigation by summarizing the results of two recent studies of professional musicians who perform on period instruments (Bangert et al., 2014, under review). The first study used experimental methods and the second took the form of a case study.

Our study of seven violinists sight-reading, practicing and performing an unfamiliar piece of solo Baroque music identified various types of decisions being made (Bangert et al., under review). The study involved tasks that were intuition-inducing (sight-reading) and analysis-inducing (thinking-aloud during practice) in order to compare the proportion of decision types in a final task of performing the piece. This design was informed by default-interventionist models of dual-process theories: sight-reading was judged to be a wholly intuitive task requiring rapid default responses to issues raised in the piece, while practice thoughts were captured through a concurrent think-aloud and asking participants to mark the score. Changes to performance features in the sight-read and performance data were analyzed, annotated, and then compared with practice data (a methodology piloted in Bangert et al., 2009). This process resulted in decisions in the final performance task being categorized as intuitive (not planned) or deliberate (planned). The study found a high percentage of intuitive decision-making, with approximately 82% of decisions made during performance categorized as intuitive. The seven participants were divided into three levels of expertise and important differences were found between these groups. Firstly, more experienced performers made a significantly greater number of decisions compared to less experienced performers. Secondly, the most experienced group made a greater proportion of deliberate decisions compared to less experienced groups.

While the study of violinists provided insights into how musicians make decisions in the early stages of learning a new piece, we have also examined how decisions are made when the performer is familiar with the musical material. In a case study of the cellist Daniel Yeadon, we were able to elicit detailed description of decision-making regarding his interpretation of J. S. Bach’s Suites for Solo Cello, BWV1007-1012 (Bangert et al., 2014). Decisions were defined as reported changes to one or more performance features and were classified according to the language used within the quotation (a methodology piloted in Bangert, 2009). Four categories of decisions were found: intuitive, procedural, deliberate, and deliberate HIP (historically informed performance).

Decisions categorized as deliberate (including deliberate HIP) accounted for 65% of the total number of decisions found, while intuitive (including procedural) decisions accounted for the remaining 35%. Intuitive decisions were based on a feeling or sense and were not explained further. In these quotations Yeadon described doing things based on “how I’m feeling at the time” (p. 41) or being “experimental” and “spontaneous” (p. 42). Deliberate decisions demonstrated conscious awareness and planning. These quotations included phrases like “I had it in my head” (p. 46) or “I’d consciously decided” (p. 47). Since the case study was of period instrument performance (Baroque cello), the category of deliberate HIP was used to highlight decisions clearly based on Yeadon’s knowledge of musical style and performing practices during the Baroque period.

An important finding from the study was the novel category of procedural decisions, a sub-set of intuitive decisions that were originally deliberate but had become automatic and “built-in” through practice over time (p. 44). The processes involved in this category resemble the concept of mature intuition proposed by Baylor (2001). For example, when discussing articulation in a certain passage, Yeadon stated, “It felt natural to me. As if I’d assimilated all the stuff I had been thinking about all those bars and I was just playing” (p. 45). Yeadon also talked about how the process of assimilation or automatization is necessary to achieve a flow state (defined by Csíkszentmihályi, 1975, 1988) during performance: “I’ve thought about what I want in each bar but when I’m actually playing I don’t really have time to nourish those thoughts and put them into practice” (p. 45). Another performer who has alluded to this process is harpsichordist Gustav Leonhardt (as cited in Sherman, 1997):

When one is a student one does things consciously, but when one is more experienced one does not play intellectually any more. One doesn’t *think*; one *has thought*. You must have done so before, but when you perform it is too late to think; you are only making music, without any thought of “now delay here” and “now articulate there” (p. 198).

In our case study, Yeadon also discussed differences between novice and expert thinking, but implied a circular motion beginning with early intuitive responses followed by a period of analysis and finally the use of intuitive/procedural decisions while “in the zone”:

How do you think your goals for these pieces [J. S. Bach’s Suites for Solo Cello] have shifted over time?

I think I’ve probably come a full circle. I think when I first played them as a kid I found it easier… I always related to the music, so I found it easier just to play them in a really instinctive way. It was just music that spoke to me and I felt that I could interpret them and be myself. Then I started studying early music and doing lots of reading of treatises, reading about how music was played at the time and what one should and shouldn’t do. It’s become a much more mental process and I’ve passed through that lens and done a lot of mental work - not on all of the suites but on some of the movements and some of the suites. In a way that’s been, the mental aspect of it, has been quite painful because it’s meant that I’ve been more self-conscious in the way I play it. I’ve been more aware of other people’s interpretations, aware of the greatness of the music. Now I’m coming back to being able to, as I was saying before, being in the zone and just making the music purely my own and heartfelt and not letting the mental stuff get in the way.

The mental work that you’ve done, do you mean performance practice research or…?

Yes, and then just thinking more carefully about the minutiae of playing, about the articulation, slurrings, dynamics, overall phrasing arches, architecture.

The quotations by Leonhardt and Yeadon both stress the use of unconscious processes in expert performance, suggesting that experienced musicians rely on intuitions informed by conscious study and what Yeadon termed “mental work.” In addition, Yeadon’s description of coming “full circle” in how he makes musical decisions provides a context for the results of the case study documented in Bangert et al. (2014). While it may feel like coming “full circle” for Yeadon in that he is relying more on intuitive processes again, the quality of his intuitions has presumably changed in line with his increased knowledge and expertise (the result of studying, reading, and “mental work”). Using Baylor’s (2001) terminology, his processes of decision-making are now guided by mature intuition (procedural) rather than the immature intuitions of a novice.

## A SPIRAL MODEL OF MUSICAL DECISION-MAKING

When incorporated with the existing theories of Evans (2011), Hammond (2007), Baylor (2001), our studies of musical decision-making raise new possibilities for modeling the role of intuition and deliberation. The review of various models related to cognitive processes, decision-making and expertise demonstrates that an individual moves through points of using more or less intuition and deliberation according to circumstances and experience. However, some recent research briefly summarized above suggests that a more comprehensive account could be developed. In particular, current models generally propose a simple movement between poles and lack representation of the unfolding process involved in the development of musical expertise and decision-making.

**FIGURE 1:**
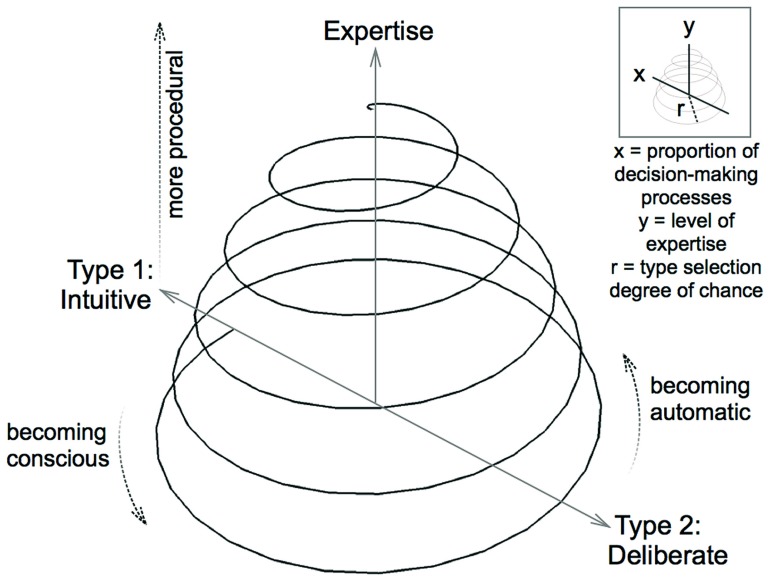
**Spiral model of musical decision-making**.

Our proposal is that the dimension of expertise development can be integrated in a new model, illustrated in **Figure 1** as a conical helix that alternates between points of more intuitive/less deliberate and more deliberate/less intuitive decision-making processes. As the upward-spiraling motion is a key characteristic of the model, we use the term spiral model in this paper to refer to the conical helix and its axes. The model that we propose extends Baylor’s (2001) U-shaped curve by acknowledging that the process of learning is a dynamic and continuous one in which a performer returns to musical problems over their lifespan with a fluctuating emphasis on either intuition or deliberation. The spiral shape and the process of action and reaction it implies has also been influenced by a similar spiral model proposed by Fabian (2003) to account for the history of expressiveness in twentieth-century music performance as evidenced on sound recordings.

The x-axis in **Figure 1** represents the proportion of decision-making processes, contrasting more intuitive (Type 1) with more deliberate (Type 2). The spiral starts from a point on the x-axis representing a primarily intuitive approach and alternates between phases of greater use of either deliberation (“becoming conscious”) or intuition (“becoming automatic”). It is important to recognize that any point along the spiral represents the use of a greater or lesser proportion of intuition or deliberation, not a sole reliance on one or the other. In other words, the x-axis in **Figure 1** is an intuitive-deliberate continuum that does not exclude contribution from either process. This acknowledges that most tasks require a mixture of Type 1 and Type 2 processes (also see Hammond’s concept of “quasirationality,” Hammond, 1996, 2010).

The spiral moves gradually upward along the vertical y-axis in **Figure 1** representing level of expertise. Based on a definition by Ericsson et al. (2007), Papageorgi et al. (2010) suggest that an expert musician is “a person who consistently demonstrates exceptional levels of performance compared to other individuals of similar age and experience and whose level of expertise can be confirmed by some form of measurable outcomes” (p. 32). Attitudes about the nature of musical expertise vary, but expert musicians are generally understood to excel across a range of musical skills such as the ability to communicate musically to an audience, practice efficiently, display technical proficiency, and so on (Creech et al., 2008). In this paper, we focus on one component of musical expertise, namely the ability to make informed decisions about performing a piece of music. Level of musical expertise has been shown to strongly relate to amounts of deliberate practice (Ericsson et al., 1993; Sloboda et al., 1996), but this is not the only factor in achieving high levels of musical proficiency (Williamon and Valentine, 2000; Hallam, 2001a; Hallam and Bautista, 2012).

The intuitive side of the spiral in **Figure 1** includes a transition toward more procedural decision-making processes signified by an upward arrow, meaning that a performer who has passed through several phases in the spiral can access procedural skills and knowledge (mature intuition). As expertise increases and more deliberate decisions become automatic, reliance on procedural processes becomes greater.

The radius of the spiral (r) in **Figure 1** is labeled type selection degree of chance, which is greatest at the base entry point and decreases incrementally as the spiral proceeds. Chance in this model is defined in opposition to control, which may be implicit or explicit (Blais, 2010). Therefore, the decreasing radius indicates that the selection of decision-making processes is more arbitrary and subject to chance in novices and that experts have greater control and consistency in how decisions are made. The tightening of the spiral in **Figure 1** also implies that the rate of changing proportions in decision-making processes becomes more rapid and relatively effortless with increasing expertise.

As the data that informs the spiral model concerns musicians engaging with a specific piece, the model is primarily conceived as a depiction of musical decision-making by an individual performer in relation to a piece over their lifespan. However, the application of the model has been purposely left somewhat open-ended. We are open to its use to explain decision-making in relation to any repertoire, either as defined in the singular or grouped by composer, style, genre, or another classifier. The time-span involved may also vary and the model could have relevance beyond the musical domain as a general theory of decision-making. In this paper we refer to pieces or repertoire rather than works or compositions as the latter terms connote greater permanency (following Leech-Wilkinson, 2012).

To take into account individual differences, we propose that the spiral in **Figure 1** need not be in a central position and could shift to any point along the x-axis. Therefore, **Figure 1** is a generalized spiral model of musical decision-making that can be varied and adapted. For example, the radius joins the y-axis in **Figure 1** due to the centralized spiral, but spirals for different performers or different repertoire are likely to have a curved or slanting vertical axis that is independent of the y-axis and determines radius.

We consider the spiral model to be a visual representation that can explain behavior and make certain predictions, but it is principally metaphorical in nature and not strictly mathematical (Gentner and Grudin, 1985; Hoffman et al., 1990). While many elements of the model could be depicted on a two-dimensional plane, we have drawn it in three dimensions to give an independent definition to changes in the radius and include the spiraling motion that will be discussed in more detail later in the paper. To further explain the model, we now describe a typical path through various points in **Figure 1** with reference to pertinent literature from psychology and music performance studies.

### MOVEMENT TOWARD GREATER DELIBERATION

Default-interventionist views of dual-process theories like Evans’ (2011) Intervention Model predict default responses to be intuitive. Following this theory, performers faced with a new repertoire or musical problem would employ primarily intuitive processes of decision-making, conceptualized as a point at the far left of the x-axis in **Figure 1**. In our study of violinists (Bangert et al., under review), the task of learning a new piece was shown to be highly reliant on intuition, even after a 45 min practice session. In both the sight-read and performance conditions, participants drew on their feelings of “rightness” or “wrongness” and a sense of what worked, what seemed natural, obvious, or appropriate to guide their decision-making. However, there was movement toward more deliberate processes of decision-making over time, particularly by experienced players who brought key issues to conscious attention and provided solutions in a more rapid, efficient manner. This is perhaps to be expected since expert performers tend to use the most efficient practice strategies (Lehmann and Jørgensen, 2012, p. 686). The difference between performers with more experience and those with less can be conceptualized in the spiral model as differences in the quality of intuitions (movement toward more procedural) and the ability to control type selection (decreasing radius).

Through analysis and deliberate practice (Ericsson et al., 1993), musicians gradually make more deliberate choices about their interpretation, moving upward from the left of the x-axis to a point on the right. This movement is labeled “becoming conscious” in **Figure 1**. The move toward deliberate decision-making processes is crucial to the ability to make informed musical decisions and is achieved by experimenting with interpretative possibilities. During practice, decisions are weighed up, reflected on, analyzed, and other options explored. Practice may be physical or mental and the strategies for planning, conducting and evaluating practice can vary considerably between individual performers (Jørgensen and Hallam, 2009). Performers may make various deliberate decisions based on a multitude of influences, such as the structure of the piece, technical challenges, knowledge of historical information, and specific past experiences. Therefore, the role of deliberation grows, but is subject to varying degrees of critical effort (Evans, 2011) according to the individual, task and context.

### MOVEMENT TOWARD GREATER INTUITION

Deliberate decisions start to become automated through practice and increased familiarity with the repertoire and musical issues being considered, moving up and back toward the intuitive side (from right to left on the x-axis). This movement is labeled “becoming automatic” in **Figure 1**.

The move from deliberation to intuition was implied in some quotations from our study of violinists (Bangert et al., under review), such as a reference to “built-in” stylistic understanding, but was most clearly articulated through the category of procedural (after Squire, 1987) found in our case study of cellist Daniel Yeadon (Bangert et al., 2014). This category was considered a subset of intuitive and consisted of deliberate decisions that had become automatic over time as the result of practice (described by Yeadon as “built-in” or “assimilated”). The categories of intuitive and procedural provide evidence for differences within Type 1 processes like Pretz’s (2011) distinction between holistic judgments that integrate complex information (termed classic or holistic intuition) and judgments based on analytical processes that have become automatic through practice (termed inferential intuition).

In the spiral model, we incorporate these differences within Type 1 processes through an upward arrow showing that performers use more procedural decision-making processes (a subset of intuitive) with increasing expertise. This is the result of the semicircular movement of deliberate decisions becoming automatic at each successive level of the spiral. Therefore, automaticity is not equivalent to intuition, but is considered a characteristic of procedural processes that gains more prominence as the spiral proceeds.

The importance of procedural processes has been raised in previous studies of music performance, including by pioneering music psychologist Seashore (1919):

One must have been intensely conscious of technique, must have known laws, must have isolated element after element for intensive study, all severely intellectual, cold, and quite free from the artistic impulse, before control of these can become so automatic as to drop into the background of consciousness (p. 259).

More recently, Chaffin et al. (2002, p. 102–107) identified a “gray” stage of practice in which the goal of the performer is to develop automaticity. In their analysis, this stage forms part of a second stage of practice within four main stages: (1) Scouting it out; (2a) Section by section; (2b) The gray stage; (3a) Putting it together; (3b) Polishing; (4) Maintenance (Chaffin et al., 2002, p. 101). Referring to expert period keyboard performers, Berkowitz (2010) describes aspects of improvised performance that are reliant on the internal ear, fingers and experience: the performer has been “trained in the basic musical building blocks to such a degree of automatization/proceduralization that these elements can be performed without conscious awareness” (p. 124).

The process of proceduralization changes how a skill is executed and the quality of intuitions more generally, resulting in the “mature intuition” described by Baylor (2001). A similar phrase from music performance studies is Rink’s (2002) “informed intuition,” a concept “which recognizes the importance of intuition in the interpretative process but also that considerable knowledge and experience generally lie behind it” (p. 36; also see Rink, 1990). This point is made more generally by Hogarth (2001), who notes that each person’s “inventory of intuitions” or “cultural capital” which they use to interpret the world is continually and mainly implicitly shaped and informed by their experiences (p. 9; also see Hogarth, 2008, p. 93–98). Experienced musicians draw on a reliable and appropriate “inventory of intuitions” for tasks or problems encountered within their domain of expertise because they have accessed and integrated relevant information and skills. In our model, “informed intuition” could simply describe the intuitions of experts, while “mature intuition” as explained by Baylor (2001) corresponds to our category of procedural (a subset of intuitive indicating deliberate decisions made automatic).

### SPIRAL MOTION

An upward spiral forms as previously deliberate decisions become intuitive and other decisions need more deliberate attention. As Moxley et al. (2012) have shown in their study of chess players, both experts and those with less skill employ and greatly benefit from extra deliberation. Musicians deliberately explore and test interpretative options, perhaps guided in their search by intuitive pattern recognition. While one or a number of options may be reinforced and made more automatic, the same decisions may be reviewed, analyzed or refined during a later phase in the spiral. The types of decisions being made may move from basic or technical considerations to interpretative or expressive features of the piece (Chaffin et al., 2002), although for performers with greater expertise, these decisions are likely to be integrated to a greater degree than with novices.

As the spiral proceeds, the radius gradually decreases to demonstrate movement from a high degree of chance in type selection to the performer having greater control. For instance, novice default responses are likely to contain guesses and mistakes and what initially becomes subject to conscious attention may be somewhat arbitrary and changeable. In contrast, experts apply and consolidate processes that are deemed appropriate and efficient for the task at hand. As Chi (2006) explains, “experts not only will know which strategy or procedure is better for a situation, but they also are more likely than novices to use strategies that have more frequently proved to be effective” (p. 24). An expert musician with high control over type selection may, for example, practice a technical challenge to automaticity, plan the interpretation of certain passages in detail, and intentionally leave another aspect to spontaneity during performance. We use the term control as not necessarily implying conscious awareness, but as a cognitive process that may be implicit or explicit (Schmidt et al., 2007; Blais, 2010).

Decisions become progressively more informed and mature as the result of the upward spiral motion until the performer is able to rely on Type 1 decision-making processes that are procedural in nature. At this stage, Type 2 decision-making processes may be used in performance to direct attention to performance cues (Chaffin et al., 2002), but much of the interpretation is “built-in.” While some performers may remain static at points in an individualized spiral, there is no endpoint in the generalized spiral in order to signify ongoing development of musical ideas, knowledge, and understanding.

Performance can occur at any point in the model, but would ideally take place when the spiral had moved upward and tightened significantly around the vertical axis. At this point, decision-making is more informed and type selection more controlled, allowing the performer to shape or direct their actions to suit the specific aims and context of the performance. By performance we mean a rendition of any kind (after Ridley, 2004, p. 111; also see Cook, 2014). Emmerson (2009) suggests that the decisions made during practice are essential, but in the end should be “transcended” in performance:

In practice and rehearsal, one plays with all sorts of variations – some obvious but many of them extremely subtle – and makes many conscious and unconscious decisions. But ultimately, most of these are in fact transcended in the act of music-making. At some point one hands the process over to the subconscious instincts to synthesize – to forge all those details into a coherent form, inevitably one that is your own (p. 117).

This quotation refers to various movements between intuition and deliberation and suggests that performance relies on procedural processes that “synthesize” decisions. Oscillation between types of thinking is also described in CCT (Hammond et al., 1987; Hammond, 2007). Like Hamm’s (1988) CCT-based study of highway engineers discussed earlier, our spiral model shows that the emphasis placed on intuition or deliberation changes over time in a non-linear way.

Other data reporting alternating periods of intuitive or deliberate thinking by musicians includes Holtz’s (2009) interview study of 17 professional musicians in which almost all participants reported “the need for an alternation between phases of deliberate construction and of intuitive experimentation” (p. 214). Similarly, Nelson and Rawlings (2007) studied eleven artists (five musicians, two writers, two visual artists, one writer/visual artist, one playwright/theater director) and found that the artistic creative processes involved movement between intuition and more critical, analytical mental processes that “may occur frequently in the course of the overall process or only several times” (p. 239; also see Allen and Thomas, 2011; Sowden et al., 2014). They explain that alternation between phases may occur for various reasons:

The artist may be prompted to become more analytical in his approach when he senses that the artwork is not “flowing” as easily and therefore loses confidence in his approach to the work; when there is a sense of elements of the artwork not “fitting together” as well as they were earlier in the process; when he encounters a technical challenge, making him contemplate how he will technically be able to convey certain ideas; or when the focus of attention moves from the artwork to another mental object (p. 235–236).

In a case study of a Western art music composer, Pohjannoro (2014) found a core compositional procedure about “deepening and extending the awareness of the substance at hand” and explained: “The procedure evolves through rapid back-and-forth movements of different intuitive and reflective compositional acts, monitored by metacognitive acts.” Drawing on dual-process theories, intuitive (Type 1) compositional acts consisted of imagination, experimentation, incubation and restructuring, reflective (Type 2) acts comprised rule-based processing, contemplating different alternatives and analytic viewing of the music, and metacognitive acts incorporated evaluation, setting musical goals (both Type 1/Type 2) and making operative plans (Type 2). In our spiral model, back-and-forth movement between Type 1 and Type 2 decision-making processes by experts is similarly mediated by a factor of control as the decreasing radius represents lessening degrees of chance in type selection.

Moment to moment oscillation between modes of thinking can occur at any time, but rapid movement by experts would correspond to a higher or theoretically ultimate point in the spiral model (highest point of **Figure 1**). In the generalized spiral, the proportion of decision-making processes appears balanced because the musician has access to both intuition and deliberation and can move between them with ease. Such an experience has been described by songwriter and guitarist Richard Thompson (as cited in Boyd, 1992):

You get inside the music to such an extent that you kind of *are* the music, or the music’s you. You’re thinking about it but you’re not thinking about it. Sometimes I think it’s almost a flashing backward and forward of intellect and intuition: One minute you’re thinking G flat, seven five, and then it’s gone and you’re doing something that you’re not aware of really (p. 162).

This description suggests an additional dimension of flow, where the performer reaches a balance between their perceived skill level and the perceived challenge and is totally absorbed in the task (Csíkszentmihályi, 1975, 1988). Csíkszentmihályi’s flow construct consists of nine dimensions, which as well as the skill-challenge balance and concentration on the task, includes a strong sense of control (Csíkszentmihályi, 1990; Jackson and Csíkszentmihályi, 1999). In our model, an increase in fluidity and control in expert musical decision-making is represented by the tightening of the spiral. A sense of control has been found to be important to flow experiences of musicians (Marin and Bhattacharya, 2013; Wrigley and Emmerson, 2013; Clark et al., 2014) and the relationship between this flow characteristic and musical decision-making warrants further investigation.

In summary, the upward-spiraling motion in **Figure 1** represents shifts in the proportion of intuitive and deliberate processes of musical decision-making over time. Performers begin by relying mainly on an immature intuition, but through deliberate processes of decision-making during practice are able to automate conscious choices, making them procedural. As performers become more expert and move up the spiral, the radius decreases to signify gradual reduction in the influence of chance and consequently, greater control (implicit or explicit) over the selection of decision-making processes appropriate for the task and performance context. Eventually, the performer reaches a point at which their use of intuition and deliberation to explore, understand, integrate and apply musical possibilities has created an ideal performance state in which the tension between the performer’s conscious and unconscious is balanced from moment to moment (Sawyer, 2012, p. 351).

## SUMMARY OF HYPOTHESES

While we have described the spiral model as a metaphorical model, its key components nevertheless lead to several hypotheses. These could be empirically tested in future research and can be summarized as follows:

(1) Musicians fluctuate in their processes of decision-making, moving alternately between phases of greater or lesser intuition and deliberation. These categories can be distinguished through observed or reported data that demonstrate characteristics of Type 1 or Type 2 thinking. Intuitive decision-making processes are fast, effortless, based on a feeling or sense, and generate default responses. Deliberate decision-making processes are slow, effortful, demonstrate conscious awareness and planning, and intervene on default responses.
(2) Novice and expert performers differ in the quality of their intuitions, the speed of their movement between phases, and their ability to control type selection. Novices access immature intuition, move more slowly between phases, and their type selection are subject to high degrees of chance. Experts can access mature intuition (procedural), move more quickly between phases, and their type selection is more controlled (implicitly or explicitly).
(3) Individualized spiral models can account for processes of musical decision-making by different performers or the same performer engaging with different repertoire. To construct individualized models, factors such as task requirements and individual differences should be taken into account. For example, an expert performer of a certain repertoire will have a tighter spiral at the base in comparison to repertoire they are not familiar with, and a performer with an intuitive cognitive style or learning strategy will spiral more along the intuitive side in comparison to a performer who favors deliberate strategies.

## LIMITATIONS AND FUTURE DIRECTIONS

The spiral model proposed in this paper provides a context for existing and future work in musical decision-making and performance science more generally. For example, the high percentage of intuitive decision-making (82%) in our study of Baroque violinists (Bangert et al., under review) and relatively low percentage of intuitive decision-making (35%) in our case study of cellist Daniel Yeadon (Bangert et al., 2014) are both best explained in the context of an ongoing, dynamic model of musical decision-making. These overall results reflect the conditions of the task and individual preferences at the time, but are likely to change as the performer develops and refines their practice and performance strategies.

By theorizing about changes to decision-making processes with increasing expertise, including the maturing of intuition, the spiral model supports the literature that makes distinctions within Type 1 processes (e.g., Pretz, 2011) and extends existing models (e.g., Baylor, 2001). However, the model is limited by data drawn from one type of music performance (notated Western art music for solo period string instruments) and by constraints of method that do not evidence the entire spiral from the novice to expert. Therefore, the general theory of a spiral movement could be tested by exploring elements that may be expected to change with increasing expertise such as the focus or content of musical decisions, the ability to articulate decisions, and the changing nature of decisions on both the intuitive and deliberate side of the spiral. Future research could also explore individual variation according to the characteristics and demands of specific tasks. Expert intuition is domain-specific and changes to the nature of the task such as the genre and style of music, composer, or level of technical difficulty would affect how the spiral proceeds over time. Therefore, the shape of the spiral and rate of movement could depend on several factors, including individual strategies and motivation, degree of existing familiarity with the repertoire, task complexity, and overall level of performer expertise.

We have addressed the issue of individual differences by proposing that the spiral in **Figure 1** may be placed anywhere along the x-axis to illustrate variation in approaches between musicians. Some performers may remain static at points on the spiral for a period of time, veer toward the intuitive or deliberate side, or dip in expertise. Thus, the general principle of a fluctuating emphasis between intuition and deliberation can be applied individually to varying degrees and lead to differing endpoints in a slanting spiral shape that is not centrally located. For example, the 65% of deliberate decisions found overall in our case study of cellist Daniel Yeadon (Bangert et al., 2014) would suggest a spiral located nearer to the deliberate pole. An influential factor on individual differences that could be investigated further is cognitive style. For example, Kreutz et al. (2008) propose that musicians process music in two ways: as systemisers engaging with formal and mechanical aspects of the music, and as empathisers demonstrating an interest in understanding the emotional and affective aspects of music. A musician with a stronger predilection for a certain cognitive style or learning strategy may be ultimately directed away from a central, balanced point in our model toward a more intuitive or deliberate approach (for relevant psychological tests see Betsch, 2008; Betsch and Iannello, 2010). Other individual differences for future study could include age, gender, instrument, musical training, and social and cultural background (Gaunt and Hallam, 2009).

A component of the model that could be investigated further is differences between novice and expert decision-making in terms of what we have termed type selection degree of chance. While decisions are always subject to chance or mistakes, we have suggested that experts have greater control (implicit or explicit) over decision-making and select Type 1 or Type 2 decision-making processes more appropriately or more intentionally to a greater degree than novices. The issue of chance and control in type selection may be affected by several factors, such as the presence or accessibility of resources (relevant knowledge and experience) or mindware (learnt rules and procedures). Differences between performers may also be partly due to metacognition, which has been found to differ between expert and novice musicians (Hallam, 2001b; Jørgensen, 2004; Bathgate et al., 2012, p. 97–98). Future research could explore how expert-novice differences in metacognitive knowledge (of task requirements and personal resources) and metacognitive control (self-regulation; Ertmer and Newby, 1996) influence the use of decision-making processes in musical tasks.

## CONCLUSION

This paper examined current understandings of Type 1 (intuitive) and Type 2 (deliberate) decision-making processes and their application to studies of music performance. We described a model intended to refine existing theories of the interaction between these processes and incorporate factors related to expertise development. Our model uses an upward-spiraling motion in the shape of a conical helix that proceeds across three dimensions with two axes (x = proportion of decision-making processes, y = level of expertise) and an ever-decreasing radius to demonstrate consolidation and control over decision-making processes with increasing expertise (see **Figure 1**). The model is based on data from studies of musicians, but could be applied to other domains. While the model incorporates elements of various theories and is supported by data outlined in the paper, further research will be required to determine the level to which predictions using the model can be verified and generalized.

## Conflict of Interest Statement

The authors declare that the research was conducted in the absence of any commercial or financial relationships that could be construed as a potential conflict of interest.
